# Improvement in Fertility and Pain after Endometriosis Resection and Adhesion Prevention with 4DryField^®^ PH: Follow-up of a Randomized Controlled Clinical Trial

**DOI:** 10.3390/jcm12103597

**Published:** 2023-05-22

**Authors:** Bernhard Krämer, Jürgen Andress, Felix Neis, Sascha Hoffmann, Sara Brucker, Stefan Kommoss, Alice Höller

**Affiliations:** Department for Women’s Health, University Hospital Tübingen, Calwerstr. 7, 72076 Tübingen, Germany; juergen.andress@med.uni-tuebingen.de (J.A.); felix.neis@med.uni-tuebingen.de (F.N.); sascha.hoffmann@med.uni-tuebingen.de (S.H.); sara.brucker@med.uni-tuebingen.de (S.B.); stefan.kommoss@med.uni-tuebingen.de (S.K.); alice.hoeller@med.uni-tuebingen.de (A.H.)

**Keywords:** clinical study, laparoscopy, adhesion prophylaxis, barrier gel, pregnancy, fertility, cycle-independent pelvic pain, dysmenorrhea

## Abstract

Background: Adhesions after endometriosis resection are frequent and the most common causes for chronic pain and secondary infertility. Primary results of our randomized controlled trial (RCT) on adhesion prevention after deep infiltrating endometriosis (DIE) resection using the gel barrier 4DryField^®^ PH showed 85% adhesion reduction in second-look surgeries. Secondary endpoint data on fertility and pain development were collected during 12-month follow-ups. Methods: This RCT comprised 50 patients. Preoperatively and after 1, 6 and 12 months, pain scores for cycle-independent pelvic pain, dysmenorrhea, dyspareunia, dyschezia, and dysuria, as well as the number of pregnancies, were recorded,. Results: The pregnancy rate in the intervention group was significantly higher (*p* < 0.05). Pain development was also improved: after 12 months, all 5 subscores were lower in the intervention group and improvements were more pronounced, most prominently concerning cycle-independent pelvic pain and dysmenorrhea, the two subcategories with the highest preoperative scores and, therefore, the highest relevance for the patients. Cycle-independent pelvic pain even recurred in the control group, while barrier application prevented this. Conclusions: Considering the known causal link between adhesions and pain, it is apparent that the favourable outcomes in the intervention group are linked to effective adhesion prevention. The significant increase in pregnancies is remarkable.

## 1. Introduction

Endometriosis affects about 10% of reproductive-age females [1]. About 70–80% of women suffering from endometriosis also suffer from associated pelvic pain symptoms [2,3], and chronic pelvic pain is a common consequence of adhesion formation after endometriosis surgery [4,5]. The four most common types of endometriosis pain are dysmenorrhea, dyspareunia, cycle-independent pelvic pain and dyschezia [2]. However, the mechanisms triggering the sensation of pain in endometriosis are still not fully understood [6]. Gibson et al. [7], for example, revealed that peritoneal macrophage and interleukin-6 abundance is correlated with the severity of pelvic pain in endometriosis. Furthermore, Chacko et al. [8] found that high magnesium intake is linked with lower levels of inflammatory markers, including interleukin-6 and tumor necrosis factor α-R2. In turn, systemic disorder in endometriosis affects magnesium transport through the cell membrane, which changes magnesium levels in peritoneal fluid in endometriosis patients [9].

Treatment options for endometriosis include medication and/or surgical resection, depending on the symptoms, lesions, desired outcome and patient’s choice [10]. Excision and ablation are the two most common techniques to resect endometriosis. Although symptomatic relief of pain can be achieved with both excision [11,12] and ablation [13], direct comparison of these two approaches did not result in significant differences in pain scores, either for mild [14] or severe cases of endometriosis [15].

When endometriosis is surgically resected, adhesion formation is a common complication. Even with a minimally invasive approach, more than 80% of patients develop adhesions after resection of deep infiltrating endometriosis (DIE) [5]. Two mechanisms of adhesions causing pain have been reported: on the one hand, adhesions contribute to pain syndromes by distorting the normal anatomy of tissues and organs. On the other hand, there appears to be a direct impact, as sensory nerve fibres, both myelinated and non-myelinated, are present in human pelvic adhesions [16]. Nevertheless, the benefit of adhesiolysis (without the application of an adhesion barrier) in patients with intraperitoneal adhesions and chronic pelvic pain is limited, and it was found that only patients with severe, vascularized and dense adhesions benefit significantly [17]. Notably, there also is adhesion reformation after adhesiolysis, and de novo adhesion formation can occur additionally [18].

Adhesion formation is also a leading cause for acquired female infertility [19]. In addition to the distortion of the pelvic anatomy, the mechanism of ovum pickup can be impaired by peritubal adhesions and ovum release. Ovulatory function can be impaired by periovarian adhesions [20,21]. For periadnexal adhesions, it was demonstrated that salpingoovariolysis resulted in a significantly improved pregnancy rate [22].

This randomized controlled trial (RCT) is a follow-up of our study on adhesion prevention after endometriosis resection and adhesion prophylaxis with a barrier: we now compare the outcome pain and fertility to the control group, testing the hypothesis that pregnancy rate and pain development are improved by using the adhesion barrier 4DryField^®^ PH as a gel.

## 2. Materials and Methods

### 2.1. Study Design

This monocentric RCT was conducted at the University Hospital for Women, Tübingen, between July 2018 and November 2019, with follow-up continuing during the subsequent 12 months. The study was approved by the Ethics Committee of the Medical Faculty at our institution (no. 217/2018BO1). It is registered in the German Clinical Trial Register (DRKS) and the International Clinical Trials Registry Platform of the World Health Organization (main ID: DRKS00014720, secondary ID: U1111-1213-4142).

A total of 50 women undergoing laparoscopic resection of endometriosis in a two-step approach were randomized into two groups. The trial was designed as a parallel study with an allocation ratio of 1. Randomization was performed using simple randomization utilizing a randomization list generated using the RAND function of Microsoft Excel. The randomization list was generated by the study nurse, who was the only person unblinded to randomization. She was present at all first surgeries, as randomization was performed during surgery. Participants were only enrolled by surgeons participating in the study, and the interventions were only carried by a small group of surgeons extensively trained in the procedures carried out in this study, who also complied with ISO 14155 (Clinical investigation of medical devices for human subjects—Good clinical practice). Patients were blinded to the group assignment as recommended by Probst et al. [23]. As both treatments in the present study are clearly distinguishable and no placebo for 4DryField^®^ PH exists, the operating surgeon automatically knew the group assignment of each patient when carrying out the treatment, thus preventing full double blinding. Additionally, the application of the analysed agent (4DryField^®^ PH vs. control) in the first surgery had to be repeated in the second intervention according to the study protocol to enable the evaluation of long-term outcomes after the second surgery. The rationale for this design is that possible adhesions formed after the second intervention would have interfered with the adhesion prevention effects after the first intervention and therefore misled the interpretation of follow-up results. Despite the general advantages of full double blinding, trials that are not fully double-blinded should not automatically be deemed inferior, and proper reporting of the blinding efforts should be considered crucial [24]. In the present study, adhesion scores were taken before the surgeon was unblinded by the study nurse for group-specific treatment at the end of each surgery. Therefore, assessment of adhesion scores was carried out while the operating surgeon and assessor was still blinded. A subsequent evaluation of adhesion scores based only on operative images or videos to enable complete blinding of the assessor was considered inferior to direct intraoperative assessment, as the evaluation of adhesion severity is based, among other criteria, on the extent of force required to lyse the adhesion following recording of the AFS score [25]. Such a subtle distinction is hardly possible based only on images or videos. Furthermore, interpretation of images or videos at a later stage carried the risk that not all areas would be documented, or that different layers of adhesions situated in rows behind each other would not be correctly interpreted and scored. This particularly applies to the extent score.

The intervention group received 4DryField^®^ PH at the end of both surgeries, while the control group received irrigation with saline solution for adhesion prevention. 4DryField^®^ PH is a starch-based powder that forms a gel after irrigation with saline solution, separating surgical sites as a physical barrier for adhesion prevention. During the second intervention, the adhesion development was scored (primary endpoint). In the second intervention, the same treatment as in the first intervention was applied following the respective study arm to ensure integrity of follow-up data. All surgeries and follow-up visits were conducted at the Department for Women’s Health at the University Hospital for Women, Tübingen. Only patients with histological diagnosis of DIE or extensive peritoneal and/or ovarian endometriosis upon diagnostic first-look laparoscopy, with the indication for a definite subsequent therapeutic procedure according to our centre’s practice (second-look laparoscopy), were included. In these patients, endometriosis was excised locally for histological confirmation and a second surgery for definite treatment was planned. The study nurse only announced group assignments afterwards, and an anti-adhesive treatment at the excision sites was carried out according to the randomization. This two-stage concept for advanced endometriosis represents the practical routine in our centre and allows optimized interdisciplinary planning concerning the extent of the condition, as well as the patient’s leading symptoms (pain, fertility issues, organ obstruction, etc.). Pregnant and/or breastfeeding patients, patients with known intolerance to starch-containing substances, and patients who did not undergo resection of endometrial tissue for histological confirmation during the first laparoscopy, and therefore did not require any adhesion-prevention treatment, were excluded. The patients were blinded during the whole study, including the follow-up.

Patient collectives and primary endpoint results of our trial are published elsewhere [26]. In summary, both groups were comparable with respect to relevant patient parameters. The results showed a statistically significant reduction of the total adhesion score by 85% in the 4DryField^®^ PH treated group (mean total adhesion score 2.2 vs. 14.2; *p* = 0.004) and the incidence of adhesion formation based on the number of affected sites was significantly reduced, by 53% (mean 1.1 vs. 2.3 sites; *p* = 0.004). No complications occurred in any of the patients during either hospital stay. Adhesions were assessed during both interventions using the same detailed adhesion score [26]. In both surgeries, 16 previously defined specific areas of interest were evaluated. The numbers of patients undergoing resections during the first surgery were as follows: right ovary, 5 in the intervention vs. 4 in the control group; left ovary, 6 vs. 5; uterosacral ligament, 7 vs. 5; round ligament of the uterus, 8 vs. 5; ovarian fossa, 4 vs. 3; right fallopian tube and broad ligament of the uterus, 0 vs. 0; left fallopian tube and broad ligament of the uterus, 1 vs. 0; uterine serosa, 2 vs. 0; rectum surface, 4 vs. 0; sigmoid colon surface, 10 vs. 8; cecal pole, 2 vs. 0; vagina, 1 vs. 0; pouch of Douglas, 8 vs. 3; psoas region, 2 vs. 1; pelvic diaphragm, 0 vs. 0; and rectovaginal septum, 2 vs. 1.

During the first surgery, the extent of possible adhesion formation sites (predilection sites) resulting from the planned endometriosis resection and/or from intended adhesiolysis was rated on a scale from 0 to 4 (0: not affected, 1: localized (less than 1/4 of the area affected), 2: moderate (between 1/4 and 2/4 of the area affected), 3: pronounced (between 2/4 and 3/4 of the area affected), 4: extensive (more than 3/4 of the area affected)) [26].

During the second surgery, the same sixteen regions were assessed for postoperative adhesions and their extent was scored in accordance with the first surgery. Additionally, the severity was scored as either 0, 1, or 4 (0: no adhesions, 1: mild (thin, avascular), 4: severe (dense, vascular)). Corresponding to the AFS score, severity and extent were then multiplied to yield a site score. The sum of all site scores added up to the total score [26].

### 2.2. Collection of Follow-up Data

There were three time points at which follow-up data was collected via telephone interviews: 1 month, 6 months and 12 months after the second surgery. In each interview, patients had to assess their pain levels with respect to cycle-independent pelvic pain, dysmenorrhea, dyspareunia, dyschezia and dysuria on a numeric rating scale (NRS) from 0 (no pain) to 10 (worst pain), with decimals also being possible. Additionally, they were asked if an adhesiolysis surgery had been recommended (and if yes, when) and if any other complications occurred. During the interview after 12 months, they were also asked if they still had the wish to conceive (if that had been their pre-surgery status) and if they had become pregnant (and if yes, when the estimated due date was). In addition to the pain scores collected during follow-up, pain scores were also taken in hospital before both surgeries for all five subcategories, and for cycle-independent pelvic pain on a daily basis after the second surgery.

**Figure 1:**
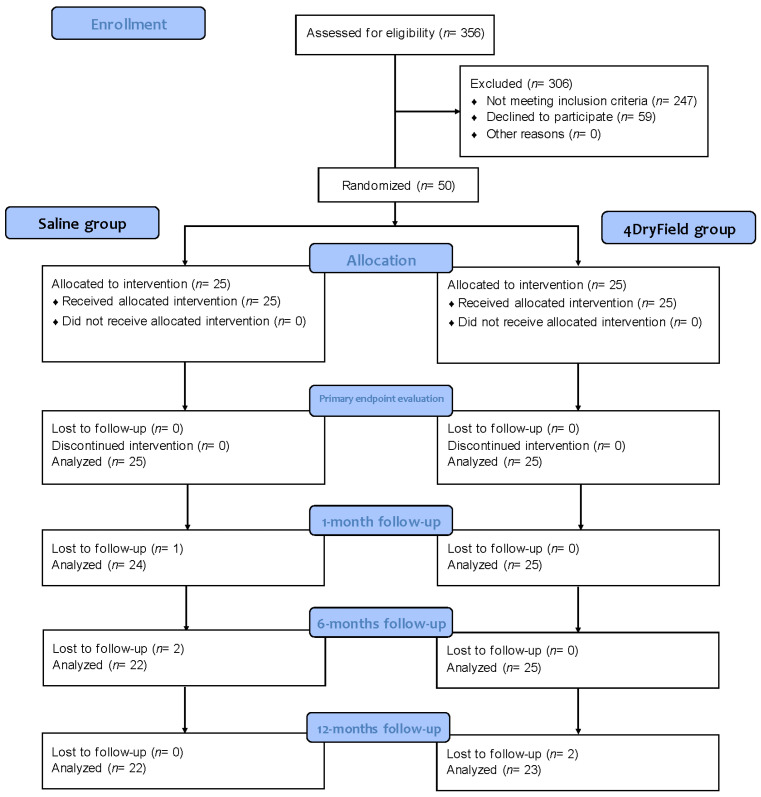
Flowchart of participants from recruitment to end of follow-up.

In total, 45 of the 50 patients (90%) completed the whole 12-month follow-up, 22 (88%) in the control and 23 (92%) in the intervention group. For patients lost at different time points during follow-up, data available from previous time points were included in the analysis (Figure 1).

### 2.3. Statistical Evaluation

Sample size determination was performed based on the primary endpoint (scoring of adhesion development) using G*Power 3.1 [27]. Results published by Korell et al. [28] and DiZeraga et al. [29] were used for this purpose. Based on these, for the control group it was assumed that 43.75% of the maximum possible adhesion score would be reached (SD: 9.375%), and 25.0% for the 4DryField group (SD: 35.35%). Combined with a one-sided *p*-value of 0.05, a statistical power of 0.8 and an allocation rate of 1, the calculation led to a required sample size of 50.

The statistical evaluation was performed using Prism 9 for Windows (GraphPad Software Inc.). Continuous variables were tested for normality of distribution. If normally distributed, unpaired *t*-tests were employed, and if not, Mann-Whitney tests were employed (both always two-tailed). To compare data within a group, paired *t*-test and Wilcoxon matched-pairs signed rank tests were used, respectively. Categorical variables were evaluated using Fisher’s exact test and time-to-event variables using the Mantel-Cox test. The level of significance was always 0.05.

## 3. Results

A total of 45 of the 50 patients completed the follow-up (90%). In the 4DryField group, two patients were lost during follow-up (92% completed) but were available for the interviews after one and six months. In the control group, three patients were lost (88% completed). One of these was not available for any of the telephone interviews, and the other two only for the interview after one month. All available data from these patients were included for the respective evaluations, leading to somewhat varying group sizes.

### 3.1. Reproductive Outcome

**Figure 2:**
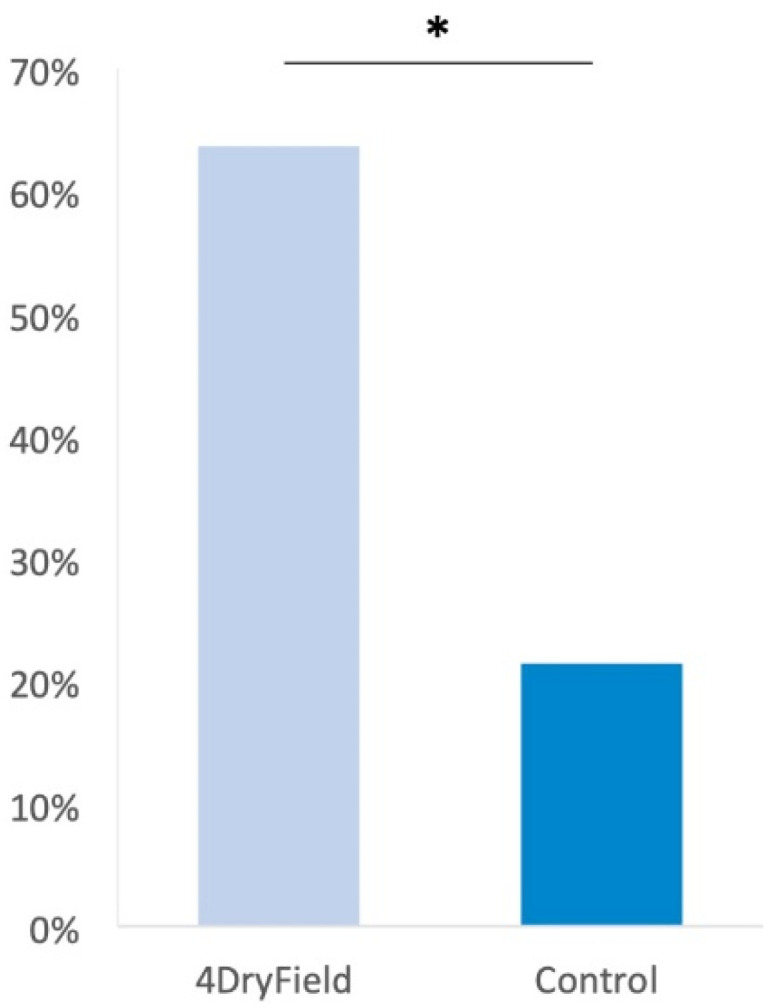
Pregnancy rates 12 months after the second surgery (*: *p* < 0.05 between the two groups).

In the intervention group, 11 patients declared and retained their wish to conceive, and 7 of these became pregnant. This corresponds to a ratio of 64%. In the control group, 14 declared and retained their wish to have a child, and 3 of these became pregnant. This corresponds to a ratio of 21%. The difference is statistically significant, with a *p*-value of 0.049. A comparison of the pregnancy rates between the two groups can be found in Figure 2.

### 3.2. Pain Development

**Figure 3:**
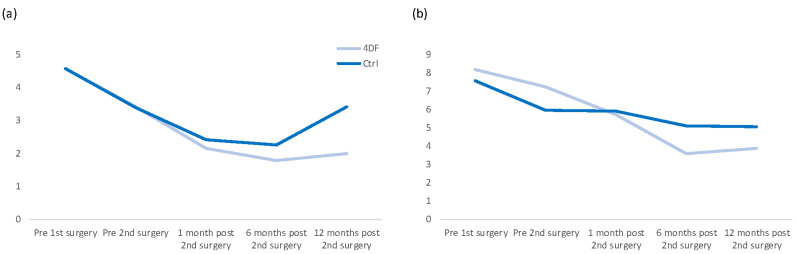
Course of the pain score development for cycle-independent pelvic pain (**a**) and dysmenorrhea (**b**). Abbreviations: 4DF = 4DryField group, Ctrl = control group.

**Figure 4:**
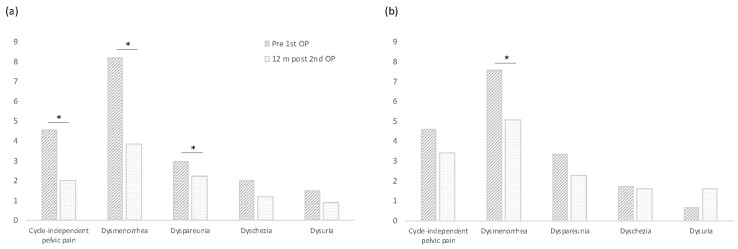
Comparison of mean pain scores before the first and 12 months after the second surgery in the 4DryField (**a**) vs. the control (**b**) groups. The difference is significant (* = *p* < 0.05) for cycle-independent pelvic pain, dysmenorrhea and dyspareunia in the 4DryField group but only for dysmenorrhea in the control group. Abbreviations: Pre 1st OP = before the first surgery, 12 m post 2nd OP = 12 months after the second surgery.

An overview of all mean pain scores is included in Table 1. The course for the two subscores with the highest pain load for the patients (cycle-independent pelvic pain and dysmenorrhea) is depicted in Figure 3. A comparison of the values prior to the first and 12 months after the second surgery for all five subscores can be found in Figure 4.

Table 2 contains *p*-values for the comparison of the pain scores before the first and at twelve months after the second, within the group as well as between groups for the two time points.

### 3.3. Complications

In the intervention group, 2 of 23 patients (8.7%) and in the control group, 3 of 23 patients (13.0%) reported having received a recommendation for adhesiolysis surgery. This difference is not statistically significant, with a *p*-value > 0.9999.

None of the patients reported any complication due to study-related treatment (no infections, wound healing disturbances, or abscesses).

## 4. Discussion

### 4.1. Main Findings

In the present study, adhesion prevention treatment led to a significantly higher pregnancy rate. The general trend was expected, as adhesions are the leading cause for acquired female infertility [19] and a distinct reduction of the overall adhesion score of 85% could be achieved. Nevertheless, the statistical significance of the difference is remarkable considering the small patient number.

Pain development was improved through the application of the adhesion barrier. After 12 months, all 5 subscores were lower in the treatment group, and the respective improvements were also higher. The effect was most prominent for cycle-independent pelvic pain and dysmenorrhea, which are the two subcategories in which the preoperative scores were highest. For cycle-independent pelvic pain, a recurrence of pain was observed in the control group, while barrier application prevented this effect (Figure 3). A direct association with postoperative adhesion formation seems likely, as pain is a typical symptom. However, the recurrence of endometriosis, which has been shown to be associated with the extent of adhesions in ovarian endometriosis [30], might also influence pain scores during follow-up.

As the rate of patients with a recommended indication for adhesiolysis surgery was also lower in the treatment group, adhesion barrier application led to better results regarding all secondary endpoints. Although statistical significance could only be reached in terms of fertility and singular pain subscores, the general trend is unambiguous and in line with the results for the primary endpoint.

### 4.2. Strengths and Limitations

It should be considered that initially the study was not powered to find significant differences concerning any of the secondary endpoints discussed here.

The perseverance of patients was rather high, with 90% of patients completing the whole follow-up of 12 months with 3 telephone interviews. This allowed substantial data collection.

The endpoint “indication for adhesiolysis surgery” has limited explanatory power, as neither the reason for the indication nor the performance and outcome of the intervention were ascertained. The physicians responsible for the subsequent care might have very different reasons for this recommendation, and data on the performance of an adhesiolysis surgery, as well as the adhesion burden observed, are not available.

Although the follow-up time of 12 months was rather short, significant improvement was found in the intervention group with respect to both fertility and pain. Although no third-look surgery was performed to assess adhesion formation after the second intervention, considering the significantly lower adhesion scores in the intervention group during the second look as compared to the control group and the generally high reformation rates of postsurgical adhesions, it is conceivable that the higher fertility rate in the intervention group is also linked to reduced adhesion formation. In general, the formation of adhesions is considered the main cause of secondary female infertility [31,32,33]. Another critical factor for pain relief and fertility is the recurrence and progression of endometriosis or its remission. In a retrospective analysis, Vignali et al. [34] evaluated the risk of recurrence of DIE after conservative surgery. Although endometriosis recurrence and progression generally have a clear impact on pain and fertility, recurrence rates of pain and clinical findings strongly differed in the follow-up of their study [34]. As recurrence of pain is linked to several further factors, particularly recurrence of adhesions [35,36,37,38], conclusions on endometriosis relapse can only be drawn based on subsequent surgery, which was not part of our study. Although our patient collective was too small to take into account all the anatomic and surgical variables potentially involved in determining the resolution of the disease and its symptoms, our cohort is still relatively large considering that only patients with histological diagnosis of DIE or extensive peritoneal and/or ovarian endometriosis upon diagnostic first-look laparoscopy, with the indication for a definite subsequent therapeutic procedure according to our center’s practice (second-look laparoscopy), were included.

### 4.3. Comparison with Other Studies

#### 4.3.1. Relationship between Pain and Endometriosis

The literature on anti-adhesive agents lacks data and comparisons on immediate clinical outcomes such as pain relief and improved fertility, which are of paramount importance for endometriosis management. Previously published randomized controlled trials on adhesion prevention after endometriosis resection and second look did not include a follow-up or endpoints other than adhesion scores [5,29,39,40]. In 2020, a Cochrane Review of barrier agents did not find any studies examining the impact on live birth rates or pelvic pain [41]. This limits the possible comparison of our results.

Crosignani et al. [42] investigated 22 infertile women who actively tried to conceive after the laparoscopic excision of severe endometriosis. The probability of becoming pregnant was 45% after 24 months, but only 18% after 12 months. This corresponds with the pregnancy rate of 21% in our control group.

Healey et al. [15] included 54 women with laparoscopic treatment of endometriosis (superficial as well as DIE) and compared preoperative pain with pain after 12 months. The aim of their study was to compare reduction of pain following laparoscopy after ablation or excision of endometriosis. In their excision group, they found pain improvements comparable to our results (Table 3).

The improvements achieved in our control group are nearly identical (dysmenorrhea and dysuria) or smaller. This might be due to only patients with DIE being included in the present study, whereas Healey et al. [15] also included patients with superficial endometriosis, who had a higher possibility of presenting with only superficial endometriosis at recurrence [43].

In a study by Abbot et al., a group of 19 women underwent laparoscopic excision of endometriosis and a re-intervention six months later, in which residual and/or recurred lesions were excised. Pain scores (on a scale from 0 to 100) gathered before and 6 and 12 months after the first surgery were: CIPP = 62 (baseline), 44 (6 months), 17 (12 months); dysmenorrhea = 78, 52, 29; dyschezia = 45; 30; 25; and dyspareunia: 65, 49, 22. This demonstrates a steady decline over time, while our data mostly show constant or even increasing values from 6 to 12 months. Abbot et al. report that at 6 months, 43% of patients had biopsy-proven endometriotic lesions, which were excised and which might be causative for the steady decline observed. This could mean that the increase we saw was (at least in part) due to endometriosis recurrence and progression, although we intended to completely excise all endometriotic tissue. However, it remains unclear if the higher pain scores in the control group were primarily due to adhesions or whether they were possibly due to endometriosis recurrences [4].

#### 4.3.2. Relationship between Pain and Adhesions

Pain after endometriosis surgery is commonly linked to adhesion formation [35,36,37,38]. Accordingly, the favourable pain development in the intervention group compared to the control group of our study might be related to an effective reduction of post-operative adhesion formation by using the adhesion barrier 4DryField^®^ PH.

Previous studies have shown direct correlations between different types of pelvic pain and adhesions [31,44,45]. In particular, strong correlations have been demonstrated for chronic/cycle-independent pelvic pain and dysmenorrhea. This corresponds with our findings in these two pain subscores.

In a study by Steege and Stout [46], patient-reported pain location corresponded with the laparoscopically identified adhesion location in 90% of patients. Furthermore, dyspareunia and pain during daily activities were significantly more likely to improve after adhesiolysis in patients without chronic pain syndrome. Cheong et al. [47] found that women with chronic pelvic pain and adhesions benefited from adhesiolysis with respect to improvement of pain and quality of life. This was also reported by other groups [48,49,50,51]. In a study of 480 patients with endometriosis by Hao et al. [37], adhesion formation was found to correlate significantly, particularly with chronic pelvic pain and dysmenorrhea, as well as dyspareunia and dyschezia. Heim [52] mentioned adhesions among the less common aetiologies for dyspareunia, and Mama [53] reported a link between inflammation, scarring and adhesions with pelvic pain, dysmenorrhea, and dyspareunia. Several further studies also found significant correlations between different types of pelvic pain, particularly chronic pelvic pain and dysmenorrhea, and adhesion formation and/or endometriosis [54,55,56,57].

Considering that a correlation between adhesion formation and pain has been shown in many studies, it is conceivable that the more favourable pain development in the intervention group is linked to an effective prevention of the adhesion development in this group as compared to the control group. In our control group, pain scores increased during the later course of the follow-up period. This observation might be explained by the general presence of adhesions, combined with the fact that the severity of adhesions increases over time [58]. Accordingly, Van der Wal et al. [59] stated that the increasing severity of adhesions over time might be an explanation for the recurrence of pain. This hypothesis is supported by the results of our study, which showed increasing pain scores in the later course of the follow-up period of the control group, particularly in cycle-independent/chronic pelvic pain. The intervention group, which showed significantly lower adhesion scores, does not show this increase in pain scores and generally exhibits more favourable pain score results, probably due to the reduction of adhesion formation by the adhesion barrier.

#### 4.3.3. Relationship between Fertility and Endometriosis/Adhesions

The negative influence of endometriosis and adhesions on fertility is much clearer than their impact on pain: chronic inflammation, tuboperitoneal distortion, hormonal changes on implantation, and decrease in ovarian reserve are relevant factors [60]. The inflammatory processes within the increased amount of peritoneal fluid noted in patients with endometriosis particularly affect oocytes, sperm transport and the embryo implantation process [60,61]. Furthermore, several studies have described adhesions as the main cause of secondary infertility [31,32,33]. Accordingly, the combination of endometriosis and adhesions constitutes a particularly severe burden for young women who wish to conceive, leading not only to serious pain but also to secondary infertility.

### 4.4. Clinical Implications

The primary endpoint results showed that adhesion prevention with 4DryField^®^ PH significantly reduced adhesion formation. The follow-up results indicate that this adhesion reduction is clinically relevant in terms of fertility and pain. So far, only a few studies on adhesion barriers have presented follow-up results, and data related to patient-reported outcomes is mostly not included. This manuscript describes data on pain relief and pregnancy rates, which can be considered as the most relevant patient-reported outcomes after endometriosis surgery.

To date, only few guidelines address adhesion prevention at all [62,63]. This is likely due to the inconclusive results achieved for most adhesion barriers in past studies [41]. However, the promising results of recent studies [26,64], as well as the continuous development of new barriers, indicate benefits and clinical needs that should be based on patient reported outcomes and favourable barrier characteristics [65].

## 5. Conclusions and Outlook

Primarily, the application of 4DryField^®^ PH gel led to an 85% reduction in adhesion scores. The clinical follow-up of this significant adhesion prevention demonstrated favourable results for the secondary endpoints of fertility and pain after endometriosis surgery. Subsequent studies with larger cohorts and a similar design are warranted to confirm our results, which could then lead to clearer recommendations regarding adhesion prophylaxis in this set of patients.

## Figures and Tables

**Table 1:** Mean values and standard deviations (SD) for all five pain scores at all five time points.

		4DryField Group		Control	
		Mean	SD	Mean	SD
Cycle-independent pelvic pain	Prior to 1st surgery	4.5	3.3	4.6	3.5
	Prior to 2nd surgery	3.4	2.7	3.4	3.3
	After 1 month	2.1	2.4	2.4	2.5
	After 6 months	1.8	2.1	2.3	2.8
	After 12 months	2.0	2.3	3.4	3.2
Dysmenorrhea	Prior to 1st surgery	8.2	2.2	7.6	2.5
	Prior to 2nd surgery	7.2	3.1	6.0	3.6
	After 1 month	5.7	3.1	5.9	3.7
	After 6 months	3.6	2.9	5.1	3.0
	After 12 months	3.9	3.6	5.1	3.5
Dyspareunia	Prior to 1st surgery	3.0	3.0	3.3	3.4
	Prior to 2nd surgery	2.3	2.6	3.0	3.2
	After 1 month	2.0	2.7	0.6	1.4
	After 6 months	1.7	2.8	1.7	2.4
	After 12 months	2.2	2.6	2.3	2.5
Dyschezia	Prior to 1st surgery	2.0	3.0	1.7	3.0
	Prior to 2nd surgery	2.2	3.2	2.0	3.3
	After 1 month	1.2	1.8	0.8	2.2
	After 6 months	0.8	2.0	1.2	2.5
	After 12 months	1.2	2.0	1.6	2.8
Dysuria	Prior to 1st surgery	1.5	2.4	0.6	1.6
	Prior to 2nd surgery	1.0	2.4	0.9	2.2
	After 1 month	0.5	1.0	1.0	2.3
	After 6 months	0.2	0.6	0.5	1.7
	After 12 months	0.9	2.1	1.6	3.1

**Table 2:** Cycle-independent pelvic pain (CIPP) (a), dysmenorrhea (b), dyspareunia (c), dyschezia (d), and dysuria (e) in both groups at the beginning and end of the study (* = *p* < 0.05).

(a) CIPP	4DryField	Control	*p*
Prior to 1st surgery	4.5	4.6	0.9835
After 12 months	2.0	3.4	0.0932
*p*	0.0024 *	0.1413	
(b) Dysmenorrhea	4DryField	Control	*p*
Prior to 1st surgery	8.2	7.6	0.32
After 12 months	3.9	5.1	0.2525
*p*	<0.0001 *	0.0009 *	
(c) Dyspareunia	4DryField	Control	*p*
Prior to 1st surgery	3.0	3.3	0.7693
After 12 months	2.2	2.3	0.977
*p*	0.0239 *	0.1761	
(d) Dyschezia	4DryField	Control	*p*
Prior to 1st surgery	2.0	1.7	0.7347
After 12 months	1.2	1.6	0.5715
*p*	0.1816	0.4062	
(e) Dysuria	4DryField	Control	*p*
Prior to 1st surgery	1.5	0.6	0.0873
After 12 months	0.9	1.6	0.7586
*p*	0.2422	0.2969	

**Table 3:** Comparison of pain score improvements after laparoscopic excision of endometriosis between our control group and previously published results. Abbreviations: CIPP = cycle-independent pelvic pain, SD = standard deviation.

	Our Control Group		Healey et al. [15]	
	Improvement	SD	Improvement	SD
CIPP	1.2	3.7	2.6	3.5
Dysmenorrhea	2.6	2.5	2.4	3.9
Dyschezia	0.9	2.7	1.8	3.5
Dysuria	0.4	2.7	0.4	2.3
Dyspareunia	−0.9	2.9	3.1	4.1

## Data Availability

All data generated or analyzed during this study are included in this published article.
